# Is the Protective Power of Vaccination Affected by the Lapse of Time?

**Published:** 1869-03

**Authors:** F. K. Bailey

**Affiliations:** Knoxville, Tennessee


CHICAGO MEDICAL EXAMINER.

N. S. DAVIS, M.D., Editor.

VOL. X. MARCH, 1869. NO. 3.

### Original Contributions.

### ARTICLE XI.

### "IS THE PROTECTIVE POWER OF VACCINATION AFFECTED BY THE LAPSE OF TIME?"

By F. K. BAILEY, M.D., Knoxville, Tennessee.

The above question is made the subject of a clinical lecture by Graily Newell, M.D., at St. Mary's Hospital Medical School.

My attention was directed to it in looking over the Examiner for November, 1863, where the lecture is copied from the *Lancet* of June 13th, 1863. This is a question which should deeply interest not only every medical man, but also all men and women who wish for themselves or their children to retain a face unspotted, or rather unpitted.

I well remember, when a child, that a man in our village had small-pox. He had taken it the "*natural way*," and, as generally is the case, died. At dead of night he was buried, and his only attendants to the grave were some of his neighbors who had had small-pox. At that time I was vaccinated; and "it worked finely;" leaving a scar, which, in after-life, was considered an indication of immunity from the terrible malady. How much it proved a protection, the sequel of this communication will tell. Very singularly, I had lived to be nearly 40 years of age, without being near enough to a case of small-pox to be exposed. Meanwhile, I had at various times introduced virus to my own arm, while vaccinating others, but without effect, except the itching known to all.

In December, 1862, while in charge of a division of the U. S. General Hospital at Quincy, Illinois, a private, belonging to the 4th Minnesota Infantry, on duty as cook, contracted small-pox. As soon as it was known that we had this disease in our wards, every one in the hospital was vaccinated, myself among the rest. The patient died on the 16th, and about the same time I was taken suddenly, and severely, sick. There was headache, and pain in the lumbar region, with a most inexcusable vomiting, which sedatives and correctives failed to allay. There was the horrible cold stage, followed with terrible heat of surface, and, in fine, all the commonly-described symptoms of variola. Still, it could not be small-pox, if vaccination, and revaccination, oft repeated, had been of any avail.

But, after more than forty-eight hours of suffering more intense than I had ever before endured, a profuse sweating commenced, accompanied by a very welcome relief from distress. On putting my hand to the edge of the hairs about the forehead, and upon the face, there were to be felt minute points, like as if small shot were imbedded in the dermis. In fact, I had varioloid most firmly developed. *Here* was a case, involving the question now under consideration. Enough had been endured to render it personally interesting. Fortunately, no other cases of either variola or varioloid occurred at this time.

There were some exposed at the time, who never had been vaccinated, as will be seen in the following. From notes now in my possession, there were then in the division seventy-one persons, who had been more or less exposed to the contagion.

Of this number, ten gave no evidence of having been vaccinated. There were no scars to be found upon their arms, nor could they remember that it had ever been attempted.

*Four* had had small-pox. Forty-five had been vaccinated before puberty, and twelve subsequent to that period. The ten unprotected persons were vaccinated, as stated above, with successful results.

Of the revaccinations, forty-eight were unsuccessful, and ten successful; one of which was upon a person who had had small-pox. By a successful revaccination is meant, a case where a scar could be found, but all the phenomena as seen in genuine original vaccination, were noticed. It is noted, also, that among the whole seventy-one individuals, there were three who had had varioloid, but they stated that they were vaccinated in early life.

During the winter of '62 and '63, no patients were sent to my division till February. The following is a copy of the report made at the end of the month:—

| | | |
|---|---|---|
| Whole No. of patients, | | 106 |
| Number who gave no evidence of having been vaccinated, | | 17 |
| " " had been vaccinated before, | | 89 |
| " " had had small-pox, | | 8 |
| " of revaccinations successful, | | 25 |
| " " vaccinations | do., | 8 |
| " successfully revaccinated who had had small-pox, | | 3 |

Nine (9) of the seventeen reported as unprotected, in whom vaccination was not successful, were subsequently carefully revaccinated, but of the result no record is at hand. A portion of them might have once been successfully vaccinated, and the evidence by scar might have been upon the lower limbs, as the arms only were examined. No cases occurred till the next December, although patients were constantly arriving. Vaccination and revaccination were carefully attended to, and monthly reports of results made, but no copies were preserved, to which reference can now be made.

On December 2d, about fifty patients were admitted, mostly from Nashville, Tennessee. Many of them had been vaccinated at different hospitals South; and some even had crusts upon the arms, not sufficiently mature to be removed.

But the orders were, that the operation should be repeated in all cases of admission, without exception, and regardless of what had been done previously. In one case, there was a crust upon the arm, and the soldier very naturally objected to the annoyance, but yielded, because it was the "*order.*" Virus was carefully inserted in the arm, near to the scab so nearly matured, and nothing more thought of the case, till in a few days he was taken with characteristic symptoms of variolous disease, followed, in due time, by a fine crop of eruption. He was at once sent to small-pox hospital, and the case proved to be one of unmodified small-pox. The eruption became umbilicated, and pits were left upon the face.

In another case, a soldier belonging to the 125th Illinois, was admitted December 2d, and, with the others, was revaccinated. Upon his arm was a large and distinctly-formed scar, and I inserted the virus upon each side of the original scar. The vaccination worked finely, and scabs formed at each point. After the crusts had begun to dry, and were nearly ready to separate, he was taken with small-pox in a very violent form, and died January 8th.

During the month of December, there were a great many cases of varioloid, characterized by febrile symptoms, and a slight eruption, but of undoubted genuineness.

Among those were many I had vaccinated and revaccinated at former periods, and it seemed evident that they were protected from the influence of contagion.

The mild cases, that required no nursing from others, were merely transferred to the fourth-story ward of the building; and as many as twenty were in that room at one time.

They were isolated merely long enough for the vesicles to dry, which seldom required more than a week or ten days. During the intense cold weather in January, 1864, the variolous disease was very malignant, and seemed determined to become general; and no one felt secure, because so many were affected who were supposed to be well protected. The young man who was detailed as prescription clerk in the dispensary had well-developed varioloid, although the virus had been inserted in his arm at several different times.

At this period, unmodified variola also prevailed in the four or five divisions of the General Hospital, as well as among the people in the city. I will mention another case, showing the potency of contagion under certain circumstances. A corporal belonging to Company "I," 72d Illinois, was a convalescent in hospital, and had been in the wards as nurse for some months. In the notes of vaccination, it is stated that he was vaccinated at the age of 15, and revaccinated twice before coming under my care. The operation was repeated by myself on his admission, with all the others, and was considered as exempt from contagion as he could well be.

Among the patients was a young man who had lost an arm; and the corporal, having the care of dressing the stump, had formed a strong attachment to him. Some time in January the young man was taken with characteristic symptoms, which proved to be a severe case of modified small-pox.

When he was sent to small-pox hospital, the corporal volunteered to accompany him as a special nurse. I objected to his going, for the reason that none were sent as nurses, except those who had had small-pox, or varioloid. But, as he had been so much exposed to severe cases that had occurred at different periods in the same ward, it was thought that he would be safe; and, as he assumed all the responsibility in case he should contract the disease, he was allowed to accompany his young comrade.

The room to which they were assigned at the pest-house was one in which a man was recovering from the disease in a severe form, and the corporal staid with both of them, night and day.

In twelve days the corporal was attacked, and had the characteristic symptoms in a severe form. The eruption came out very thickly, and the case was one of well-developed modified small-pox.

This, we see, was an instance where the person had withstood the contagion in a less degree of intensity for some time, but succumbed after remaining constantly in a room with the disease. The corporal soon recovered; and it would seem that he could, after such an experience, be considered exempt, if any one ever could. During this period, there were three or four cases of well-developed varioloid in persons whose faces were already pitted, and had, as well as all the rest, been revaccinated under the general order. When a Teuton or a Celt, who, in the Old Country, had had small-pox from inoculation, became subjects of varioloid, our faith in such a thing as immunity was considerably shaken. Late reports from California state that serious doubts are entertained upon this subject in that region. One thing is very conclusively shown, which is, that epidemic influence will, in this disease now under consideration, as well as some others, render all rules and laws liable to exceptions.

It is very evident that people generally are becoming thoughtlessly indifferent to vaccination in their families. We frequently hear, when it is suggested to a mother that her children should be vaccinated, the exclamation, "Why! is the small-pox about?" When informed that it is not, but liable to occur at any time, she will say, "Then I will wait, for I don't want the little thing to be sick."

The late war found great numbers of men who stated that they were vaccinated in childhood, "but that it did not work," and the operation was never repeated. But very few had been revaccinated, and consequently, in cases where the original operation, although successful, had "run out," they were fit subjects for variola, especially when prevailing as an epidemic. Had they remained at home in their rural neighborhoods, they might never have been exposed.

One reason of the indifference of people upon the subject of being protected from variolous contagion is, that but few have ever seen a case of small-pox. One walk through the rooms of a "*Pest-House*" (which every one, of all things, will avoid) would suffice to cause alarm, and awake attention to vaccination.

In conclusion, then, it may be remarked, that even if vaccination is not an absolute immunity from variola, still, the disease is so much modified, that fatal results seldom follow.

The case mentioned above, of the soldier in the 125th Illinois, is the only exception, in some hundreds of individuals, during an observation extending over a period of nearly three years.

As a result of these observations, it is clear, that if every person could be vaccinated in infancy, revaccinated at the age of puberty, and at the age of twenty-one, there will be a degree of immunity as nearly absolute as can be conceived.

But, while one can have varioloid without serious results to himself, still, the mildest form is capable of communicating to an unprotected person the disease in an unmodified and fatal type. The above observations are given, in the main, from facts only deduced from memory. If access could be had to official reports made from month to month, many more cases might be presented of a similar nature.

Others may have observed the same facts; but, if so, they have not given publicity to the results of their observations.

---

